# Mapping the patchwork: Exploring the subnational heterogeneity of child marriage in India

**DOI:** 10.1016/j.ssmph.2020.100688

**Published:** 2020-11-16

**Authors:** Lotus McDougal, Holly Shakya, Nabamallika Dehingia, Charlotte Lapsansky, David Conrad, Nandita Bhan, Abhishek Singh, Topher L. McDougal, Anita Raj

**Affiliations:** a Center on Gender Equity and Health, University of California San Diego, 9500 Gilman Drive, MC 0507, San Diego, CA, 92093, USA; b United Nations Children's Fund (UNICEF), UNICEF House 3, United Nationsl Plaza, New York, NY, 10017, USA; c International Institute for Population Sciences, Govandi Station Road, Deonar, Mumbai, 400 088, India; d Kroc School of Peace Studies, University of San Diego, 5998 Alcala Park, San Diego, CA, 92110, USA

**Keywords:** Child marriage, India, Norms, Geospatial, Spatial Durbin model, Hot spot

## Abstract

Despite dramatic reductions in child marriage over the past decade, more than one in four girls in India still marry before reaching age 18. This practice is driven by a complex interplay of social and normative beliefs and values that are inadequately represented in national- or even state-level analyses of the drivers of child marriage. A geographic lens was employed to assess variations in child marriage prevalence across Indian districts, identify hot and cold spots, and quantify spatial dependence and heterogeneity in factors associated with district levels of child marriage. Data were derived from the 2015-16 National Family Health Survey and the 2011 India Census, and represent 636 districts in total. Analyses included global Moran's I, LISAs, spatial Durbin regression and geographically weighted regression. This study finds wide inter- and intra-state heterogeneity in levels of child marriage across India. District levels of child marriage were strongly influenced by geographic characteristics, and even more so by the geographic characteristics of neighboring districts. Districts with higher levels of female mobile phone access and newspaper use had lower levels of child marriage. These relationships, however, were all subject to substantial local spatial heterogeneity. The results indicate that characteristics of neighboring districts, as well as characteristics of a district itself, are important in explaining levels of child marriage, and that those relationships are not constant across India. Child marriage reduction programs that are targeted within specific administrative boundaries may thus be undermined by geographic delineations that do not necessarily reflect the independent and interdependent characteristics of the communities who live therein. The geographic, social and normative characteristics of local communities are key considerations in future child marriage programs and policies.

## Highlights

• Child marriage prevalence is highly heterogeneous across Indian districts.
• Districts near state border districts tend to have higher levels of child marriage.
• Characteristics of districts and their neighbors influence child marriage.
• Geography affects the relationship between social normative factors and child marriage.
• Child marriage interventions may require joint action across states.

## Introduction

Despite global recognition that child marriage violates the health and human rights of girls, no world region is projected to meet the Sustainable Development Goal of eliminating this practice by 2030 (Raj, 2010; Efevbera, Bhabha, Farmer, & Fink, 2019; UNICEF Data and Analytics Section, 2018). India is home to more than 15 million women aged 20–24 who married below the age of 18, the largest number of any nation in the world, and is therefore a priority country in which to understand and address this abuse (Population Division of UN Department of Economic and Social Affairs (UNDESA), 2017; International Institute for Population Sciences (IIPS) & ICF, 2017). Over the past decade, India has recorded a nearly 50% reduction in the prevalence of child marriages, from 47% in 2006 to 27% in 2016 (International Institute for Population Sciences (IIPS) & ICF, 2017; International Institute for Population Sciences (IIPS) and Macro International, 2007). This reduction has occurred contemporaneously with improvements in girls’ education, social participation, and status, as well as a legal prohibition against marriage before age 18 (International Institute for Population Sciences (IIPS) & ICF, 2017; International Institute for Population Sciences (IIPS) and Macro International, 2007; Government of India, 2007). Nonetheless, more than one in four girls in India still marry before age 18, with girls residing in rural areas and in socially and economically disadvantaged states, such as Rajasthan, Uttar Pradesh, and Bihar, at greater risk for early marriage (International Institute for Population Sciences (IIPS) & ICF, 2017).

Child marriage is driven by normative values regarding the status, value and rights of women and girls, as well as economic, social, legal and safety considerations (Raj, 2010; McDougal et al., 2018; Raj et al., 2019; Jha, Minni, Priya, & Chatterjee, 2016; Lee-Rife, Malhotra, Warner, & Glinski, 2012). Research from around the world has revealed a complex nexus of factors that contribute to this practice (Bicchieri, Jiang, & Lindemans, 2014; Fenn, Edmeades, Lantos, & Onovo, 2015; Islam, Haque, & Hossain, 2016; UNICEF-UNFPA, 2019). While poverty and educational status are strongly associated with child marriage, in many parts of South Asia and Africa, child marriage is encouraged through cultural traditions that dictate gender-discriminatory norms rooted in patriarchal values and ideologies (Shakya et al., 2018; Cislaghi et al., 2019b). Research from India and Niger suggests that girls who marry young are less likely to have a say in the choice of who they marry (Shakya et al., 2020; Santhya et al., 2010), and that in areas with low levels of gender equality, the age of marriage tends to be lower (Desai & Andrist, 2010).

In such contexts child marriage often occurs in a social framework in which cultural models enforce an idea of womanhood that is strongly associated with purity, modesty, submission to men and to elders, and the importance of motherhood. Parents in these settings often see no motivation to postpone marriage past puberty. By marrying off a daughter at a young age, a family is able to protect the honor of the family, and of the child herself, while ensuring that she attains the economic security and respectable status afforded to a married woman (McDougal et al., 2018). Parents also perceive that girls who are married earlier are at lower risk for sexual violence, and less likely to engage in premarital sexual activities (Verma, Sinha, & Khanna, 2013), despite more than a decade of evidence from India highlighting greater risk for sexual violence in marriage for those married as minors (Raj et al., 2010; Begum, Donta, Nair, & Prakasam, 2015). Community norms can counter advancements in knowledge to sustain local and family beliefs that child marriage is socially protective (McDougal et al., 2018). These localized norms may contribute to the continuation of the practice of child marriage in certain states and communities in India, even as the prevalence of child marriage has declined for the nation as a whole.

In recent years, development and public health interventions to address child marriage have increasingly focused on the role of social norms, emphasizing community engagement as well as social and behavior change strategies to shift these norms at the community level (UNFPA & UNICEF, 2018a). While such efforts recognize the contextual nature of social norms as they function at this local level, there is a need for a broader understanding of how factors associated with child marriage, and their relationships with each other, vary across social and geographic contexts. Such variation suggests that the social normative influences that contribute to child marriage transcend simple sociodemographic associations, and require consideration of “place” (Akinyemi et al., 2015; Islam et al., 2016; Maiga et al., 2015; Shiferaw et al., 2015).

Spatial demographers consider place to be an important determinant of attitudes and behaviors. An obvious reason is that geographic features can inhibit or facilitate behaviors due to commonalities such as structural access to resources (for instance distance to a health clinic). More importantly, perhaps, place is important because it is through spatial clustering of socially connected individuals that clustering of social norms typically occurs (Weeks et al., 2004, 2015). It is well known that people with similar sociodemographic characteristics typically choose to interact with each other, a concept known as homophily (McPherson, Smith-Lovin, & Cook, 2001). However because of shared exposures, social reinforcement and direct social influence, people who are geographically proximal to each other can also *become more alike*, a concept known in spatial analysis as *spatial dependence*. Spatial dependence is the phenomenon whereby people living and interacting in close proximity to one another are more likely to mutually influence each other's behaviors, and therefore characteristics, than they are the behaviors of those who live at a greater distance (Weeks et al., 2015). *Spatial heterogeneity* (also known as spatially varying relationships), on the other hand, occurs when relationships between different characteristics or behaviors change according to geographic context (Weeks et al., 2015). In other words, spatial heterogeneity takes a relationship between certain characteristics or behaviors that might normally be quantified at only a single, more aggregate level (e.g. a national-level estimate), and identifies how that relationship may vary according to place (e.g. the same relationship may manifest differently in different villages, communities or districts). The existence of spatial dependence and/or spatial heterogeneity can be important markers for patterns of social norms dispersion and variation across geographic communities, providing information that can inform social norms change intervention strategies.

In norms theory, a person's reference group is the group of people to whom an individual turns for expectations around appropriate behavior (Cislaghi & Heise, 2018). Ideally, reference groups would be identified through the use of explicitly measured social network ties (Shakya et al., 2014; Shakya et al., 2015), but in almost all health and development research, such data are lacking. As an alternative, researchers can generate data with behavioral or attitudinal measures across more crude social units from which social ties can be inferred, such as across residents of the same village or neighborhood to determine whether there is inter-cluster variation. High levels of variation across these spatial units can be viewed as evidence of variability in norms, and may indeed capture collective, or community, norms (Mackie et al., 2015; Shakya et al., 2019; Costenbader, Lenzi, Hershow, Ashburn, & McCarraher, 2017; Lapinski & Rimal, 2005; Sedlander & Rimal, 2019; Shulman & Levine, 2012). This same principle holds true for spatial heterogeneity. As an example, if the association between education and child marriage varies by region, this suggests that there may be geographically-specific social effects that are driving the behavior beyond what is considered the average association between those two factors (Weeks, Getis, Hill, Agyei-Mensah, & Rain, 2010). Often, for pragmatic reasons, child marriage programs are implemented based on administrative units such as state, district, village, or neighborhood, which may or may not match the actual contours of the participant communities. A better understanding of the role of place and other spatial/geographic dimensions in shaping and upholding social norms can help to better tailor child marriage interventions and social and behavior change strategies to the specific communities of interest.

The prevalence of child marriage, the social and normative factors associated therewith, and the programs and policies designed to intervene therein, are not uniform across India. National, and even state-level, analyses of these factors likely mask district-level inequities (Liang et al., 2019; Roest, 2016; Srinivasan et al., 2015). Not only are states across India quite distinct from one another in a variety of aspects, including population, geography, economy, religion, and culture, but programs designed to mitigate child marriage are generally implemented at smaller scales (International Institute for Population Sciences (IIPS) & ICF, 2017; Jha et al., 2016; Jacob, 2015; Dheer, Lenartowicz, & Peterson, 2015; Prakash et al., 2019; Harriss, 1999). This study thus responds to the global call for an increased understanding of intra-national differences in child marriage levels (Anonymous, 2015). This geographic analysis of child marriage is designed to explore subnational variations in the prevalence of child marriage and the social and connectivity factors that may influence child marriage norms in India. The specific objectives are to assess geographic variations in child marriage across Indian districts, identify hot and cold spots, and quantify spatial dependence and spatial heterogeneity in factors associated with child marriage. Understanding how the social and normative factors that influence child marriage cluster, disperse and interact differently within and across different geographies highlights place-based variations that may directly contribute to improved targeting of social and behavior change prevention efforts.

## Methods

*Data sources.* Non-geographic data were obtained from the fourth National Family Health Survey (NFHS-4) and the 2011 India Census, cross-sectional, nationally representative datasets collected in 2015-16 and 2011, respectively (International Institute for Population Sciences (IIPS) & ICF, 2017; Census of India, 2011). Indian district maps from 2011 were obtained from ML Infomap (ML Infomap, 2011). District population density was derived from the 2011 India Census, district distance to state borders and district area were derived from the district map shapefiles, and all other variables were derived from NFHS-4.

*Measures*. The outcome of interest was district-level prevalence of child marriage, defined as marriage or cohabitation before age 18, excluding marriages in which gauna was not performed (i.e. spouses do not co-reside and the marriage has not been consummated, which was 0.5% of this sample) among women aged 20–24 years (International Institute for Population Sciences (IIPS) & ICF, 2017).

Covariates were selected to represent the social and normative factors most related to child marriage in the available data. Geographic measures were included to account for the physical characteristics of each district, and comprised log distance from each district to the nearest non-ocean state or union territory border (per 100 km; hereafter referred to as ‘non-ocean state border’ for brevity), log district area (km^2^) and log district population density (population/km^2^). Ocean borders were excluded from distance calculations, as distance to the nearest border was intended to measure the distance to a distinct administrative, legal, and therefore economic context (Harriss, 1999). In the context of India, as in many other countries, distance to border may also serve as a proxy for environments that tolerate or even produce illegal practices, such as child marriage (Government of India, 2007). This is due to reduced capacity of local government to police trans-jurisdictional activities and actors, as well as to disparities in local laws and, consequently, prices of related goods and services (Idler, 2019; Reno, 2013; Su, 2018).

Sociodemographic variables associated with child marriage specifically, and gender inequities more broadly, were selected based on previous research (Raj et al., 2015, McDougal et al., 2018, Raj et al., 2019; Roest, 2016; Srinivasan et al., 2015). These measures included the percentage of women aged 15–49 in the district residing in rural areas, the percentage of women in a district who identified as members of scheduled castes, scheduled tribes or other backwards classes (SC/ST or OBC; legally recognized groups of marginalized individuals), the percentage of women in a district who identified as Muslim, the mean years of education among women aged 20–24 in the district, the ratio of all female to all male births to district residents in the past 6 years (per 1000), and the difference between the district vs. state levels of child marriage (positive numbers indicating districts with prevalences higher than the state prevalence). Measures of connectivity were included to assess exposure to media messages and normative discussion about child marriage and the status of women and girls. Media connectivity variables included the percent of women aged 15–49 in the district who report watching television, listening to the radio or reading the newspaper at least one per week (respectively), the percent of women age 15–49 in the district who have a mobile phone that they use, and the percent of households in the district that have internet access. Community connectivity was measured by the percent women age 15–49 in the district who were aware of microcredit programs in the area, and who reported using microcredit programs. Microcredit program awareness and utilization serve as proxy measures for community connectivity in this context, as the majority of microcredit programs in India follow a Self-Help Group model, in which participants support one another and work collaboratively to problem-solve (Batra, 2012; Folgheraiter & Pasini, 2009; Sankaran, 2005).

*Analyses*. The unit of analysis was the district, which is an administrative sub-division within Indian states or union territories. As analyses focused on proximity-based spatial relationships, districts with no neighbors (e.g. islands) or missing data were excluded (5 of 641 districts), for a total of 636 districts. There were an average of 18 districts in each state or union territory (range 1–71). We conducted both global and local exploratory and confirmatory geographic analyses. Exploratory analyses were used to calculate the prevalence of child marriage and each covariate across assessed districts. Global Moran's I was calculated for each variable. Moran's I is a global measure of spatial dependence, or autocorrelation, that measures whether the distribution of a given variable is random across geographic units (districts), or if that distribution is influenced by other nearby units (Getis, 2008). As Moran's I is a global measure, it provides a single statistic for the entire group of analyzed units (again, districts) that indicates whether, across all analyzed districts, the distribution of a given variable is geographically random or not. Moran's I test statistic and p-values were computed using Monte Carlo simulations. Distinct from (global) Moran's I, local indicators of spatial autocorrelation (LISA) measure the degree of non-random spatial clustering around a specific unit (district) (Anselin, 1995). The local Moran's I statistic is calculated as a LISA, with “hot spots” classified as spatially clustered districts with significantly similar higher values of a given variable, and “cold spots” classified as spatially clustered districts with significantly similar lower values of a given variable. P-values of <0.05 were used for all cluster identification.

Multivariable regression analyses explored spatial variations in two ways. First, global confirmatory spatial analysis used a spatial Durbin regression model to assess multivariable associations while accounting for spatial dependence. Spatial Durbin regression was selected a priori due to its ability to generate unbiased effect sizes, and its flexibility with regards to spillover effects (Elhorst, 2010; LeSage & Pace, 2009). Additionally, model fit robustness checks were conducted using likelihood ratio tests to compare nested regression models (spatially lagged X, spatial lag model, spatial error model, spatial Durbin error model, spatial Durbin model). The optimum fits across these models were the spatial Durbin model, and spatial Durbin error model (results not shown). As these two models are not nested in one another, and thus could not be compared using likelihood ratio tests, AIC was used as a final indicator of model fit, indicating that the spatial Durbin model was the best fit (AIC of 3381.7 vs. 3403.9). Spatial Durbin models include a spatially lagged dependent variable as well as spatially lagged independent variables, and can be represented as follows:

$$y=\rho Wy+X\beta +WX\theta +\alpha 1_{n}+\varepsilon$$

where y is the dependent variable (district-level prevalence of child marriage), *ρWy* is the spatial autoregressive effect, or the endogenous effect of other districts' dependent variable on a given district's dependent variable, (*ρ* being the spatial autoregressive parameter, *W* being a row-standardized, queen contiguity spatial weight matrix representing the influences of neighboring districts, and *Wy* being the spatially lagged dependent variable [district-level prevalence of child marriage]), *Xβ* is the vector of independent variables, *WXθ* is the vector of spatially lagged independent variables (again using *W* as the spatial weight matrix representing the influences of neighboring districts), *α1*_*n*_ is the intercept coefficient for a vector of 1 by n geographic units, and *ε* is the error term. (Elhorst, 2010; LeSage & Pace, 2009) Spatially lagged variables are thus contiguity-weighted averages of that variable's values in neighboring geographic units. As spatial Durbin models are able to assess global and local spillover effects that may vary across independent variables, (Anselin, 1988; Elhorst, 2010; LeSage & Pace, 2009) they produce simulated estimates of direct, indirect and total effects. Direct effects are those in which changes in the vector of independent variables in a given unit (district) change the dependent variable in that unit. (Elhorst, 2010; LeSage et al., 2014; LeSage & Pace, 2009) Indirect effects, often termed spatial spillovers, measure the degree to which changes in the vector of independent variables in a given unit (district) change the dependent variable value in other units (districts). Total effects are the sum of direct and indirect effects.

Second, geographically weighted regression illustrates how global effects may vary across space (spatial heterogeneity). This approach applies linear regressions locally to each unit of analysis (districts) to show how the relationships between factors associated with child marriage may vary in different geographic areas (Fotheringham, Brunsdon, & Charlton, 2002). All variables used in the spatial Durbin model were fitted to a multivariable linear regression model that was then applied locally at each district to calculate district-level regression coefficients, represented as follows:

$$y_{i}=\;\beta_{i0}+\sum_{k=1}^{p}\beta_{ik}x_{ik}+\varepsilon_{i}$$

where *y* is the dependent variable (district level prevalence of child marriage) in district *i*, *β*_*i0*_ is the intercept term in district *i*, *p* is the number of independent variables, *β*_*ik*_ is the regression coefficient for independent variable *k* in district *i*, *x*_*ik*_ is independent variable *k* in district *i,* and ε_i_ is the error term in district *i*. Geographically weighted regression results are mapped showing significant (p < 0.10) coefficient results calculated from t-values. (Mennis, 2006)

All analyses were conducted in Stata 15.1 and R 3.6.0. Ethical exemption for analysis of this publicly available, deidentified data was provided by the University of California San Diego.

## Results

The prevalence of marriage before age 18 among women age 20–24 across assessed Indian districts in 2015-16 is 25% (Table 1). On average across districts, female residents are mainly rural-residing (72%), identify as SC/ST or OBC (75%), have 9 years of education, and television is the most common form of regular media connectivity reported (69% weekly use). An average of more than one in three (37%) women across districts were aware of microcredit programs, and an average of one in six (16%) women across districts reported using a microcredit program. All assessed variables showed significant spatial dependence, indicating that the levels of assessed variables were not randomly distributed across districts. The magnitude of this spatial dependence ranged from a high of 0.75 (female weekly television use) to a low of 0.31 (sex ratio at birth), indicating that all variables have non-random, spatial clustering that warrants adjustment.

**Table 1:** Descriptive statistics for assessed variables.

	Mean (SD)	Global Moran's I	
		Test statistic	p-value
Child marriage prevalence (%)	25.3 (13.7)	0.71	0.01
**Geography**			
Log distance to state border (per 100 km)	8.2 (0.9)	0.46	0.01
Log density (population/km^2^)	−1.0 (1.2)	0.69	0.01
Log area (km^2^)	15.1 (1.0)	0.47	0.01
**Sociodemographics**			
Rural residents (%)	71.6 (21.5)	0.43	0.01
SC/ST or OBC (%)	74.7 (20.3)	0.65	0.01
Muslim (%)	12.6 (17.4)	0.74	0.01
Female education (years)	9.0 (2.1)	0.70	0.01
Female:male sex ratio at birth among births in the last six years (per 1000)	902.5 (59.9)	0.31	0.01
District-state differences in prevalence of child marriage (%)	0.6 (9.4)	0.35	0.01
**Media connectivity**			
Female weekly television use (%)	68.7 (22.1)	0.75	0.01
Female weekly radio use (%)	10.3 (9.2)	0.68	0.01
Female weekly newspaper use (%)	24.3 (14.5)	0.64	0.01
Female mobile phone access (%)	45.6 (16.9)	0.66	0.01
Household has internet access (%)	13.1 (12.3)	0.64	0.01
**Community connectivity**			
Female microcredit program awareness (%)	37.4 (17.6)	0.54	0.01
Female microcredit program utilization (%)	15.9 (12.5)	0.44	0.01

SD = standard deviation.

**Fig. 1:**
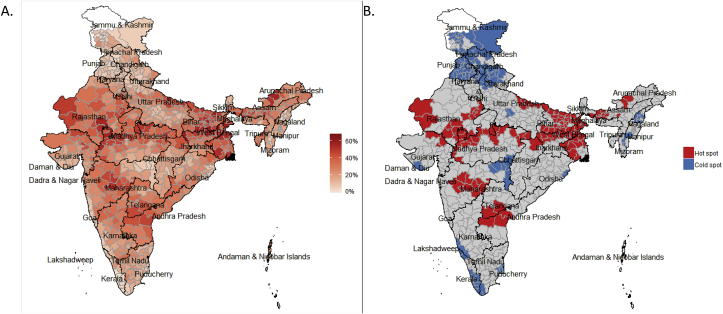
Prevalence of child marriage among women aged 20–24 years across districts (A), and local indicators of spatial association (B) in 2015-16. Note: In Fig. 1B, hot and cold spots indicate clusters of districts with high and low (respectively) child marriage prevalences that are statistically similar to their neighbors at p < 0.05. Grey indicates no significance and white indicates data not available.

District-level mapping shows wide inter- and intra-state heterogeneity in levels of child marriage (Fig. 1). Hot and cold spots (clusters of districts with high and low levels of child marriage, respectively) appeared concentrated around state borders, with examples of this seen for the border of Bihar and Jharkhand, Rajasthan and Madhya Pradesh, and Telangana and Andhra Pradesh. Spatial clustering was also clear for levels of female education, with clusters of lower levels in the eastern and western regions, and clusters of higher levels of education in the northern and southern regions (Appendix Fig. 1). Media use tended to be clustered in the northern and south-western parts of the country, with central India having clusters of low mobile phone access. In terms of community connectivity, districts with higher levels of microcredit awareness and utilization were clustered in southern and south-eastern India.

Multivariable spatial Durbin modeling indicates that district-level child marriage prevalences are driven largely by direct effects from sociodemographic and connectivity measures, and by both direct and indirect geographic effects. Districts that were farther from non-ocean state borders had lower levels of child marriage, though these associations were driven by indirect effects, often referred to as spillovers (−5.5, p = 0.001). In other words, districts *near border-proximate districts* tend to exhibit higher levels of child marriage. Overall, increases in both log population density and log area were associated with increases in child marriage prevalence; the magnitude of association was larger for indirect effects (9.3, p < 0.001 and 3.5, p = 0.02, respectively) than direct effects (1.7, p < 0.001 and 1.4, p < 0.001, respectively) (Table 2).

**Table 2:** Spatial Durbin multivariable regression model assessing associations with district-level child marriage prevalence in India, 2015–16.

	Direct marginal effects				Indirect marginal effects				Total marginal effects			
	Coefficient	SE	z-value	p-value	Coefficient	SE	z-value	p-value	Coefficient	SE	z-value	p-value
**Geography**												
Log distance to non-ocean state border (per 100 km)	0.170	0.200	0.804	0.421	−5.502	1.686	−3.259	0.001	−5.332	1.761	−3.028	0.002
Log density (population/km^2^)	1.363	0.289	4.755	<0.001	3.551	1.464	2.412	0.016	4.914	1.498	3.275	0.001
Log area (km^2^)	1.735	0.256	6.699	<0.001	9.339	2.338	4.003	<0.001	11.075	2.497	4.436	<0.001
**Sociodemographics**												
Rural residents (%)	−0.009	0.014	−0.579	0.562	−0.080	0.091	−0.808	0.419	−0.089	0.098	−0.829	0.407
SC/ST or OBC (%)	−0.031	0.012	−2.455	0.014	−0.136	0.076	−1.830	0.067	−0.167	0.080	−2.106	0.035
Muslim (%)	−0.023	0.017	−1.345	0.179	−0.093	0.092	−0.967	0.334	−0.116	0.097	−1.147	0.251
Female education (years)	−0.596	0.157	−3.820	<0.001	−3.052	0.894	−3.473	0.001	−3.684	0.940	−3.941	<0.001
Female:male sex ratio at birth (per 1000)	−0.004	0.003	−1.283	0.200	−0.003	0.027	−0.199	0.843	−0.007	0.029	−0.320	0.749
District-state differences in prevalence of child marriage (%)	0.768	0.019	39.623	<0.001	−0.133	0.151	−0.931	0.352	−0.636	0.161	3.898	<0.001
**Media connectivity**												
Female weekly television use (%)	−0.016	0.017	−0.949	0.343	−0.167	0.100	−1.692	0.091	−0.183	0.104	−1.781	0.075
Female weekly radio use (%)	0.022	0.026	0.790	0.429	0.037	0.164	0.249	0.803	0.059	0.169	0.364	0.716
Female weekly newspaper use (%)	−0.120	0.022	−5.420	<0.001	−0.004	0.125	0.028	0.978	−0.124	0.130	−0.890	0.373
Female mobile phone access (%)	−0.043	0.017	−2.537	0.011	0.181	0.107	1.677	0.094	0.139	0.113	1.207	0.227
Household has internet access (%)	−0.011	0.022	−0.510	0.610	−0.114	0.123	−0.977	0.329	−0.125	0.130	−1.010	0.312
**Community connectivity**												
Female microcredit program awareness (%)	0.014	0.011	1.288	0.198	−0.077	0.075	−1.018	0.309	−0.062	0.081	−0.771	0.441
Female microcredit program utilization (%)	0.075	0.015	5.002	<0.001	0.163	0.118	1.419	0.156	0.237	0.126	1.919	0.055
**Spatial effect**												
Rho (Spatial lag)									0.85			<0.001
**Model fit statistics**												
AIC									3380.8			
Nagelkerke pseudo R^2^									0.94			
Log-likelihood									−1655.38			
Lagrange multiplier test (residuals' autocorrelation)									15.02			<0.001

Results show distributions based on 500 multivariable normal distribution simulations. Fit statistics (AIC, R^2^, log-likelihood, LM test) are for the entire model.

Districts with higher levels of women who self-identified as SC/ST or OBC had slightly lower levels of child marriage (−0.03, p = 0.01) (Table 2). Female education was an important factor associated with lower levels of child marriage in terms of direct (within-district) associations, as well as the spillover effects of neighboring districts on levels of child marriage (Table 2). For every additional year of average female education in a district, there was a 0.6% reduction in the prevalence of child marriage in that district. For every additional year of average female education in neighboring districts, there was a 3.1% reduction in the prevalence of child marriage in a given district. District-state differences in child marriage levels were positively associated with direct effects on child marriage prevalence, but not indirect effects. Rural residence, the percent of Muslim female residents, and sex ratio at birth were not associated with district levels of child marriage in this model.

In terms of media connectivity, the majority of effects were direct. The prevalence of women reading a newspaper weekly was associated with a direct effect decrease in child marriage prevalence (every 10 percentage point increase in weekly newspaper use was associated with a 1.2 percentage point decrease in the district prevalence of child marriage) (Table 2). Districts with higher female mobile phone access had lower levels of child marriage (Table 2). Every 10 percentage point increase in female mobile phone access was associated with a 0.4 percentage point decrease in child marriage prevalence. Female television use was indirectly, though marginally, associated with child marriage. For every increase of 10 percentage points in female weekly television use in neighboring districts, there was a 1.7 percentage point reduction in child marriage prevalence in a given district (p = 0.09). Female radio use, household internet access and microcredit program awareness were not associated with district levels of child marriage in this model. Microcredit program utilization, however was associated with a slight increase in district child marriage levels (for every 10% of the female population who reported microcredit program utilization, there was a 0.8 percentage point increase in district child marriage levels).

**Fig. 2:**
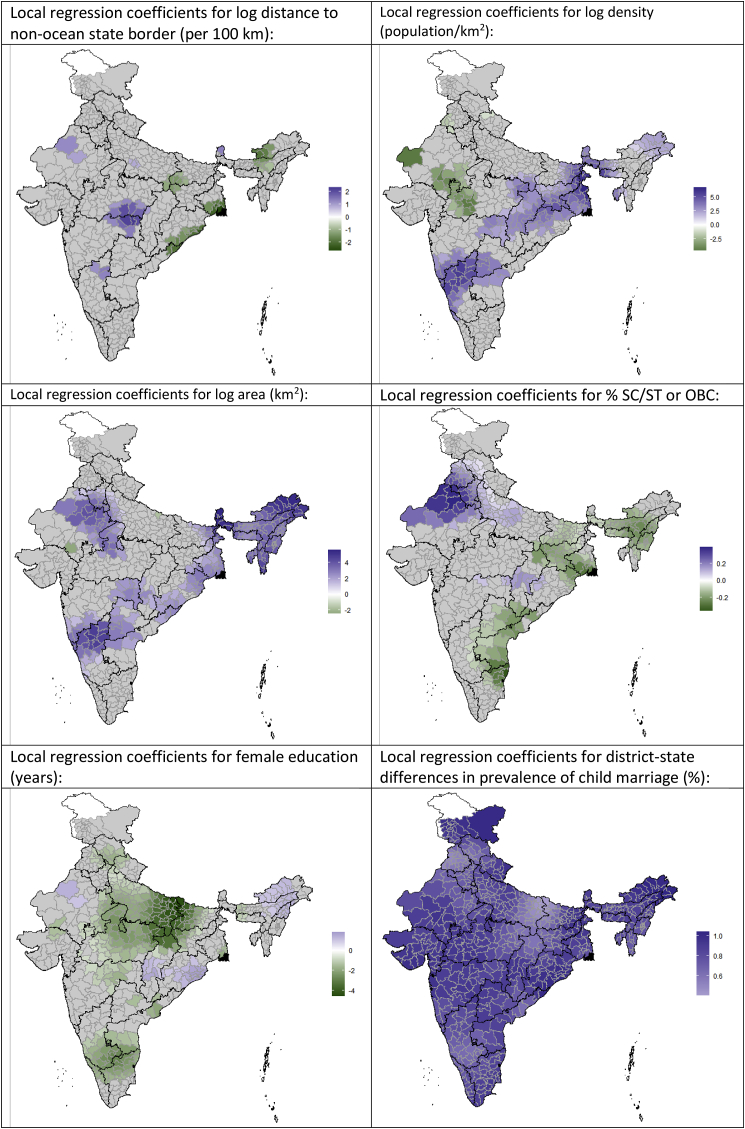

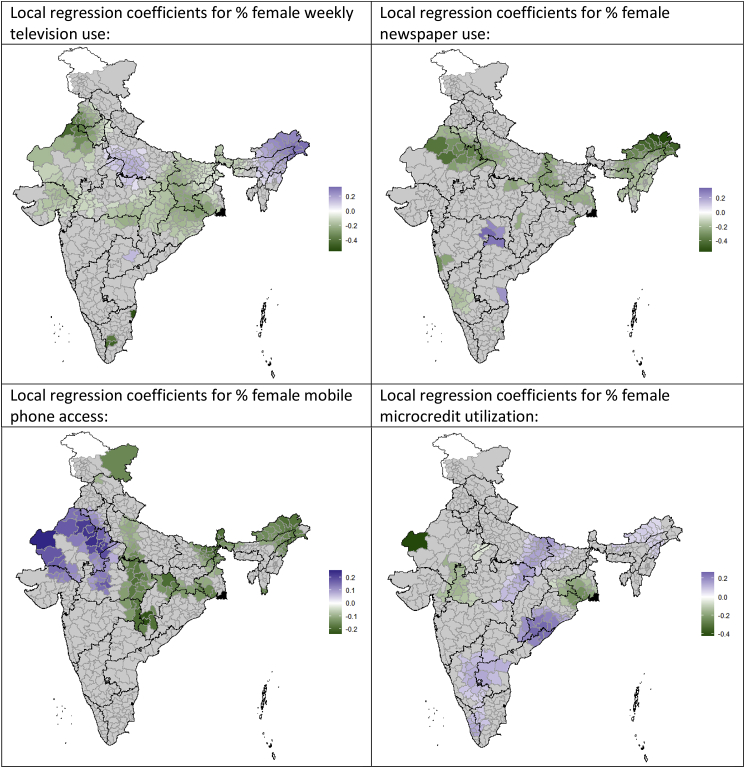
Geographically weighted regression showing local regression coefficient values for the association between predictor variables and levels of child marriage across Indian districts, 2015–16. Regressions are adjusted for all variables shown in Table 2; only coefficients significant at p < 0.10 are displayed.

Geographically weighted regression was used to explore local spatial heterogeneity in the associations identified through the global spatial Durbin model. All variables that were significant in Table 2 showed substantial heterogeneity in their coefficients of association with district-level child marriage levels (Fig. 2). The district-specific associations between distance from non-ocean state borders and levels of child marriage were most strongly positively clustered around the borders between Madhya Pradesh, Chhattisgarh and Maharashtra (e.g. longer distances to state borders were associated with higher district levels of child marriage), and most strongly negatively clustered in West Bengal, Odisha and Arunachal Pradesh (e.g. longer distances to state borders were associated with lower district levels of child marriage). The associations between higher population density and higher prevalences of child marriage were strongest in a swath of districts from the south-west of India to the north-east, spanning a number of states. SC/ST or OBC status was most associated with higher prevalences of child marriage in northern Rajasthan, and most associated with lower levels of child marriage in northern Tamil Nadu and southern West Bengal. Associations between higher levels of female education and lower levels of child marriage were strongest around the Uttar Pradesh/Bihar/Chhattisgarh border areas. Television use was most strongly associated with lower levels of child marriage in Tamil Nadu and northern Rajasthan, while newspaper use was most associated with lower levels of child marriage in Arunachal Pradesh, central Rajasthan, and the Uttar Pradesh/Bihar/Jharkhand border area. Female mobile phone access was associated with lower levels of child marriage in central and north-eastern India, particularly in southern Chhattisgarh and eastern Maharashtra.

## Discussion

Findings from this study underscore the need for a nuanced understanding of the geographic, cultural, social, normative and political contexts that influence child marriage in India, and emphasize the importance of local factors associated with child marriage which may be masked by national, or even state-level, estimates. This analysis of the first National Family Healthy Survey in India designed to be representative at the district level reveals substantial subnational geographic variation in the district-level prevalence of child marriage among women aged 20–24 in India, as well as the presence of spatial dependence and spatial heterogeneity in factors associated with child marriage. This indicates both that the relationships between some district-level sociodemographic and normative factors (e.g. marginalized group status, female education and some forms of media access) and child marriage levels are influenced not only by the prevalence of those factors within each district, but also levels of those factors in neighboring districts. Additionally, the strength of those relationships varies substantially across geographies.

Geographic factors were strongly predictive of child marriage in multivariable regression accounting for that spatial dependence, suggesting that these place-based characteristics (particularly density and area) are strongly related to levels of child marriage within communities (districts, in this analysis). This relationship was influenced not only by the district's own characteristics, but also by the characteristics of neighboring districts. This statistically significant interplay across districts' characteristics highlights the tremendously contextual nature of child marriage, and particularly the social and normative factors that influence age at marriage (Cislaghi et al., 2020). Hotspots of heightened levels of child marriage were identified in border districts of Madhya Pradesh, Rajasthan, West Bengal, Bihar, Jharkhand, Andhra Pradesh, and Telangana.

These relationships between neighboring states are of great importance in understanding the sub-national heterogeneity of child marriage in India. While Indian states may differ from one another with respect to administrative capacity and legal frameworks (Harriss, 1999), many state boundaries can be considered porous in terms of culture, with people from the same caste or sub-caste communities residing in adjacent states (Dheer et al., 2015). In the context of child marriage, this is key, as traditionally, marriages in India occur between individuals from the same sub-caste or community (Kaur & Palriwala, 2018). In states with male-skewed sex ratios like Rajasthan, families have been known to buy brides from neighboring states, with the majority of the girls being minors (Times of India, 2017). While, to the authors’ knowledge, no academic studies examine the spatial prevalence of this practice, it is plausible that such transactions are more common across border regions. Indeed, we find evidence that there are significant and negative indirect effects of distance to state borders, demonstrating that districts whose neighboring districts are near state borders generally exhibit higher prevalences of child marriage. These results seem to substantiate the idea that the illegal practice of child marriage clusters in state border areas, and that additional, geographically-focused prevention efforts may be warranted.

The sharing of communities across state borders may be of particular importance for recently formed states based on shared laws, histories and cultural practices. Telangana and Andhra Pradesh were a single state prior to 2014, with people from the same communities inhabiting both states, clustered around the border regions (Srinivasulu, 2002). Jharkhand was a part of Bihar prior to 2000, and shares borders with both Bihar and West Bengal. There are hot spots of child marriage along all of these recently formed borders. Tribal child marriage fairs are relatively common in districts of West Bengal that are bordering with Jharkhand, and see participation from all neighboring regions (Jha et al., 2016). The neighboring states of Jharkhand, West Bengal, Bihar and Uttar Pradesh also face issues with the trafficking of minor girls across the India-Nepal border, a risk factor for child marriage in that families may marry their minor daughters due to fear of sexual violence (Deb & Sanyal, 2018; Verma et al., 2013; Vindhya & Dev, 2011). These more recently-formed border areas also tend to share similar characteristics in terms of sociodemographic (e.g. SC/ST or OBC) and media/communication (e.g. television and newspaper use in the Bihar/Jharkhand border area) factors associated with child marriage.

This area is also the region in which direct, and particularly indirect, effects of district population density drive child marriage levels. Population density of neighboring districts was identified as a strongly positive predictor of child marriage prevalence overall, and spatial heterogeneity analyses found this relationship to be particularly strong in areas of Karnataka, Chhattisgarh, Jharkhand and West Bengal. Some of the geographically-specific characteristics that may influence this relationship in these areas include high percentages of marginalized groups, low levels of female education (see Appendix Fig. 1), generally low population densities and few large cities apart from Hyderabad. Urbanization in such a context may confer wealth but fewer social norm shifts often associated with urbanity (Elias & Jephcott, 1994). Income distributions between less and more densely sectors are dynamic: relatively wealthy rural residents tend to urbanize, but may find themselves less wealthy and occupying lower social status than their new urban cohorts (Datt, Ravallion, & Murgai, 2016), especially when urban development has been prioritized in national planning (Fan, Chan-Kang, & Mukherjee, 2005). As few megacities exist in this geographic corridor, urbanization may thus magnify the social normative practices of the sending areas. These trans-border cultural, ethnic and social and normative commonalities stand in relatively stark contrast with the majority of child marriage interventions, which are often still organized with states as the unit of analysis and implementation (Government of India, 2020; Jha et al., 2016; Ministry of Women and Child Development and Government of India, 2010); this geographic disconnect may limit the effectiveness of these programs, with particular consequences for communities near state borders.

Higher levels of female education in a given district were significantly associated with lower levels of child marriage, in line with many previous studies (Raj et al., 2019; Baird, Chirwa, McIntosh, & Özler, 2010; Kalamar, Lee-Rife, & Hindin, 2016; Lee-Rife et al., 2012; Malhotra, Warner, McGonagle, & Lee-Rife, 2011). Importantly, however, the results also identify a significant *indirect* association between female education and child marriage, one that is in fact substantially larger in magnitude than the direct effect. High levels of female education in neighboring districts predict lower levels of child marriage in a given district five times more powerfully than those within its own boundaries. Thus, while the level of female education within a district has a strong and negative association with child marriage, that association is substantially larger based on levels of girl education in neighboring districts. Cross-border marriages, including the practice of bride buying noted above, may in part explain this finding. An additional plausible explanation is that of social diffusion, where districts with higher levels of girl education and lower levels of child marriage may be more likely to both passively and actively share those values and norms, as well as backlash and stigma associated with norm divergence, with nearby communities (Nguyen et al., 2019; Starmann et al., 2018). This may be indicative of a broader social benefit mechanism to girl education, highlighting positive spillover effects of the normalization of increased valuation of girl education and gender equity and thus increased emphasis on girls' schooling and delayed marriage. In India, child marriage prevention programs have in large part focused on incentives for completing secondary school, though there have also been initiatives to improve girls’ empowerment, autonomy and rights awareness (Jha et al., 2016; Kalamar et al., 2016; Lee-Rife et al., 2012; Mehra, Sarkar, Sreenath, Behera, & Mehra, 2018; Prakash et al., 2019). Implementation of most of these programs, even those that are centrally funded, has generally been targeted to select states or districts, and evidence is mixed on their effectiveness (Government of India, 2020; Jha et al., 2016; Kalamar et al., 2016; Lee-Rife et al., 2012; Ministry of Women and Child Development and Government of India, 2010; Prakash et al., 2019). These results suggest that interventions designed to reduce child marriage through education would be well served by considering regional, rather than targeted, interventions to increase education in a broader geographic area that may bridge across state borders.

Media connectivity, as measured via weekly newspaper use and mobile phone access for women, was strongly associated with lower district levels of child marriage, even after accounting for other covariates; this association was present, but marginal, for television use. Access to these forms of media may be broadly representative of higher socioeconomic status, which tends to be associated with lower levels of child marriage (Raj, 2010; Efevbera et al., 2019). Indeed, 74% of women in the highest household wealth quintile in India have mobile phone access, compared to only 22% in the lowest quintile; 61% of women in the highest wealth quintile report weekly newspaper use, vs. 5% in the lowest quintile (International Institute for Population Sciences (IIPS) & ICF, 2017). The associations between media connectivity and child marriage, however, had heterogeneous coefficient values across India, suggesting that there are geographically-specific factors differentially affecting these relationships. To the extent that child marriage prevention programming uses newspapers or text messages as part of their communication strategies, this variation may reflect differences in programming availability and messaging across India. The fact that radio use was not associated with child marriage in this analysis may reflect its low overall prevalence (only 11% of women nationally reported weekly radio use), which may simply be too small to influence norms and behaviors in the nation as a whole (International Institute for Population Sciences (IIPS) & ICF, 2017).

As a result of the normative underpinnings of child marriage, prevention programs, as with many initiatives focused on behavior and norms change, commonly use multi-channel social change communication strategies: individual, small group, or community mobilization approaches within the local community that directly target program recipients, while mass media conveys advocacy, education and awareness messages more broadly to reinforce interpersonal communications (Gage, 2013; Jha et al., 2016; Kalamar et al., 2016; Lee-Rife et al., 2012; Mehra et al., 2018; Prakash et al., 2019; Svanemyr, Amin, Robles, & Greene, 2015; Wakefield, Loken, & Hornik, 2010). The use of mass media creates opportunities for program messages to reach beyond direct intervention recipients, thus expanding and amplifying the potential reach and effects of the program. Diffusion of this sort has been seen in many programs aiming to shift norms and behaviors, as well as within child marriage prevention programs in diverse settings including India, Bangladesh, and Ethiopia (Amin, Saha, & Ahmed, 2018; Gage, 2013; Mehra et al., 2018; Nguyen et al., 2019; Starmann et al., 2018). Connectivity, including to one's neighbors and communities, is one of the main ways that these messages can spread, and there is evidence to suggest that these diffusion pathways can be explicitly integrated into program design (Cislaghi et al., 2019a; Starmann et al., 2018).

However, the associations between weekly newspaper use, mobile phone access and child marriage were primarily direct, and not reflective of neighboring districts. This suggests that diffusion is either occurring at a more localized level (within districts, rather than across), that diffusion of the association between media connectivity and child marriage is not yet uniformly occurring across India, or as noted above, that media connectivity may be indicative of socioeconomic status. Indeed, the geographically heterogeneous associations between media connectivity and child marriage identified in this study highlight stronger protective relationships in the East and Northeast regions. This suggests that the multi-channel approach engaged in social and behavior change efforts is not a universally effective approach across India, but must be contextualized to locally relevant media modalities; newspaper and mobile phone channels may merit particular consideration.

These associations between media connectivity and child marriage should particularly be considered in terms of reach and relationship (International Institute for Population Sciences (IIPS) & ICF, 2017). While newspapers offer a unidirectional transfer of information, mobile phones are more bidirectional, enabling users to initiate and receive communications with others, as well as to receive information in the case of smartphones (not assessed in these data). On average, mobile phones are able to reach more Indian women than most forms of traditional mass media, barring television; overall, districts had nearly twice as many women with mobile phone access compared to those who read the newspaper weekly (46% vs. 24%). However, women with media and mobile phone access both tend to live in urban areas, be more educated, and live in wealthier households, suggesting that these approaches risk missing the most marginalized and vulnerable populations (International Institute for Population Sciences (IIPS) & ICF, 2017). As mobile phones become more and more common across India, particularly among younger women, they offer important opportunities for regular access to populations both at risk of, and with influence over, child marriage. These avenues for communication may offer low-cost means for engaging more young people in conversations around child marriage messaging and creating additional opportunities for youth-centered interventions and change. Communities with higher levels of mobile phone access for women may be better positioned to benefit from mobile phone-focused child marriage programming efforts, but the relationships between mobile phone access and early marriage in those communities should be considered when determining the suitability of this programmatic approach. Additionally, caution is needed, as the most vulnerable girls and women may be least likely to have access to this technology, emphasizing the importance of closing the digital divide.

Only one measure of community connectivity was associated with child marriage in these analyses. In multivariable models, higher levels of female microcredit utilization were associated with slightly increased levels of child marriage. This finding should be interpreted in context, and with caution. It is not an indication that microcredit programs increase risk for child marriage in India; these data are cross-sectional, and represent average levels of microcredit program participation among all women in India. Microcredit programs can serve as indicators of area level deprivation, as they are often targeted to women who are more socially and economically compromised (Swain & Wallentin, 2009). The prevalence of these programs may thus be acting as a marker of risk for child marriage rather than a direct measure of community connectivity. Importantly, measures of microcredit program awareness and participation were included in only a subset of interviews, and designed to be representative at the state, rather than district, level. The authors, some of whom were involved in the design and implementation of NFHS-4, believe these data were of adequately high prevalence for limited inferences to be made at the district level, but these findings should be interpreted with caution. Additional measures of community connectivity, which is a complex concept, would be of benefit to explicate this relationship and are unfortunately not available in these data.

Effective child marriage programmatic adaptation to local contexts requires both a detailed understanding of those contexts, as well as buy-in and effective, coordinated local partnerships (Chandra-Mouli, Plesons, Barua, Sreenath, & Mehra, 2018; Jha et al., 2016; Lo Forte, Plesons, Branson, & Chandra-Mouli, 2019). These partnerships are needed at the local district level, but also as a convergence of efforts by neighboring districts and states in order to effectively address the practice of child marriage. This further illustrates the importance of community engagement and multimedia efforts that address communities, even when dispersed across state borders, as well as the need for coalitions of districts that cross state borders, as opposed to efforts that are nationally driven or even state-based. Examining factors associated with child marriage from a geographic perspective that accounts for the complex ways that place influences this practice is an important lens with which to advance understanding of the ways that norms affect child marriage at community, rather than individual, levels. Additionally, these findings suggest that traditional markers of geographic administrative boundaries may be inadequate, and even counter-productive, in identifying which communities and localities most influence social norms around child marriage. Place-based engagement efforts that call on officials in neighboring localities to work in concert are likely to be more effective. Further, participatory approaches that engage communities in identifying and drawing their own boundaries of community identification and social network connections can be particularly informative in defining programming areas (Bates, Marvel, Nieto-Sanchez, & Grijalva, 2019; Igras, Diakite, & Lundgren, 2017).

This study must be interpreted in terms of its limitations. All self-response survey data are subject to recall bias and social desirability bias. As data were aggregated to the district level, this is an ecological analysis, and results should not be interpreted to be representative of each individual living within assessed districts. Data are cross-sectional, and causality cannot be presumed. Finally, this study concerns itself with norms and social change communications with imperfect proxies. For example, one may broadly measure media utilization, but details are lacking of that utilization source and content (e.g. community vs. state vs. national media, child marriage programming exposure). Nevertheless, these data represent the best available measures of these phenomenon in a recent, nationally-representative Indian sample.

Even as significant gains have been made in reducing the rates of child marriage in India at the national level, intra-state inequalities highlight areas where greater push may be needed to enforce policies to more uniformly prevent this practice. Gender discriminatory norms are reinforced by a lack of educational and economic alternatives to child marriage, especially for girls in the lowest socioeconomic strata. These risks are likely to compound in the context of the COVID-19 pandemic with many countries, including India, facing school closures and economic distress (Svanemyr et al., 2015; UNFPA & UNICEF, 2018b; United Nations, 2020). Identifying areas of increased vulnerability to COVID-19-related child marriage accelerations may allow policy-makers to recognize intra-state disparities and more directly target the needs of vulnerable districts. Because of the complexity of factors contributing to child marriage, efforts to combat it span many domains. Prior research has called for understanding of structural factors as well as norms that influence child marriage (Roest, 2016); this analysis supports this call, but additionally demonstrates that geographic place, and the characteristics of neighboring geographies that broadly represent culture and normative values, matter for levels of child marriage and key associated factors. Given the heterogeneous array of child marriage prevention programs that have been implemented in India, in terms of target population and program content and design, these findings underscore the need for a deeper understanding of local challenges to roll-out, delivery, and uptake (Jha et al., 2016; Kalamar et al., 2016; Lee-Rife et al., 2012). Mass media such as newspapers and mobile phones, may be important communication avenues for child marriage prevention and norm change programs, but additional research into the influence of locally rooted, place-based forms of media (i.e. community media) would likely offer additional insights into the most influential platforms for community engagement, particularly in districts near border-proximate districts. Program designers should also consider that girls’ education is important within local contexts (districts, in this case), but even more so within larger spatially-dependent cultural and socioeconomic contexts in which normative behaviors and exchange systems associated with child marriage thrive. Finally, more research is needed to understand differential risks for child marriage among border-proximate districts that may require joint action by states. Identifying reasons for these heightened vulnerabilities is needed to facilitate and improve intra- and cross-state child marriage prevention efforts.

## Financial disclosure

This research was funded by grants from the 10.13039/100000865Bill and Melinda Gates Foundation (OPP1179208, PI: Raj) and 10.13039/100006641UNICEF (20160656, PI: Raj).

## Ethical statement

Ethical exemption for analysis of this publicly available, deidentified data was provided by the University of California San Diego.

## Declaration of competing interest

LM, HS, ND, NB, AS, TLM and AR have no declarations of interest. CL and DC are employed by one of the agencies that funded this analysis. Their input into analyses and objective interpretation of findings were not influenced by their employment.

## References

[bib1] Akinyemi A., Adedini S., Hounton S. (2015). Contraceptive use and distribution of high-risk births in Nigeria: A sub-national analysis. Global Health Action.

[bib2] Amin S., Saha J.S., Ahmed J.A. (2018). Skills-building programs to reduce child marriage in Bangladesh: A randomized controlled trial. Journal of Adolescent Health.

[bib17] Anselin L. (1988). Spatial econometrics: Methods and models.

[bib18] Anselin L. (1995). Local indicators of spatial association - Lisa. Geographical Analysis.

[bib19] Baird S., Chirwa E., McIntosh C., Özler B. (2010). The short-term impacts of a schooling conditional cash transfer program on the sexual behavior of young women. Health Economics.

[bib20] Bates B.R., Marvel D.L., Nieto-Sanchez C., Grijalva M.J. (2019). Community cartography in health communication: An asset-based mapping approach in four communities in rural Ecuador. Journal of International and Intercultural Communication.

[bib21] Batra V. (2012). The state of microfinance in India: Emergence, delivery models and issues. Journal of International Economics.

[bib22] Begum S., Donta B., Nair S., Prakasam C.P. (2015). Socio-demographic factors associated with domestic violence in urban slums, Mumbai, Maharashtra, India. Indian Journal of Medical Research.

[bib23] Bicchieri C., Jiang T., Lindemans J.W. (2014). A social norms perspective on child marriage: The general framework.

[bib24] Census of India (2011). Census data. http://www.censusindia.gov.in/2011-Common/CensusData2011.html.

[bib25] Chandra-Mouli V., Plesons M., Barua A., Sreenath P., Mehra S. (2018). How can collective action between government sectors to prevent child marriage be operationalized? Evidence from a post-hoc evaluation of an intervention in Jamui, Bihar and Sawai Madhopur, Rajasthan in India. Reproductive Health.

[bib26] Cislaghi B., Denny E.K., Cisse M. (2019). Changing social norms: The importance of "organized diffusion" for scaling up community health promotion and women empowerment interventions. Prevention Science.

[bib13] Cislaghi B., Mackie G., Nkwi P., Shakya H. (2019). Social norms and child marriage in Cameroon: An application of the theory of normative spectrum. Global public health.

[bib27] Cislaghi B., Heise L. (2018). Four avenues of normative influence: A research agenda for health promotion in low and mid-income countries. Health psychology: Official journal of the division of health psychology.

[bib15a] Cislaghi B., Nkwi P., Mackie G., Shakya H. (2020). Why context matters for social norms interventions: The case of child marriage in Cameroon. Glob Public Health.

[bib28] Costenbader E., Lenzi R., Hershow R.B., Ashburn K., McCarraher D.R. (2017). Measurement of social norms affecting modern contraceptive use: A literature review. Studies in Family Planning.

[bib29] Datt G., Ravallion M., Murgai R. (2016). Growth, urbanization, and poverty reduction in India.

[bib30] Deb H., Sanyal T. (2018). Human trafficking: An overview with special emphasis on India and West Bengal. IOSR Journal of Humanities and Social Science.

[bib31] Desai S., Andrist L. (2010). Gender scripts and age at marriage in India. Demography.

[bib32] Dheer R.J.S., Lenartowicz T., Peterson M.F. (2015). Mapping India's regional subcultures: Implications for international management. Journal of International Business Studies.

[bib33] Efevbera Y., Bhabha J., Farmer P., Fink G. (2019). Girl child marriage, socioeconomic status, and undernutrition: Evidence from 35 countries in sub-Saharan Africa. BMC Medicine.

[bib34] Elhorst J.P. (2010). Applied spatial econometrics: Raising the bar. Spatial Economic Analysis.

[bib35] Elias N., Jephcott E. (1994).

[bib36] Fan S., Chan-Kang C., Mukherjee A. (2005). Rural and urban dynamics and poverty: Evidence from China and India.

[bib37] Fenn N.S., Edmeades J., Lantos H., Onovo O. (2015). Adolescent pregnancy and family formation in West and Central Africa: Patterns, trends and drivers of change.

[bib38] Folgheraiter F., Pasini A. (2009). Self-help groups and social capital: New directions in welfare policies?. Social Work Education.

[bib39] Fotheringham A.S., Brunsdon C., Charlton M. (2002). Geographically weighted regression: The analysis of spatially varying relationships.

[bib40] Gage A.J. (2013). Child marriage prevention in Amhara region, Ethiopia: Association of communication exposure and social influence with parents/guardians' knowledge and attitudes. Social Science & Medicine.

[bib41] Getis A. (2008). A history of the concept of spatial autocorrelation: A geographer's perspective. Geographical Analysis.

[bib42] Government of India (2007). The prohibition of child marriage act, 2006.

[bib43] Government of India (2020). Beti Bachao Beti Padhao. https://wcd.nic.in/bbbp-schemes.

[bib44] Harriss J. (1999). Comparing political regimes across Indian states: A preliminary essay. Economic and Political Weekly.

[bib45] Idler A. (2019). Borderland battles: Violence, crime, and governance at the edges of Colombia's war.

[bib46] Igras S., Diakite M., Lundgren R. (2017). Moving from theory to practice: A participatory social network mapping approach to address unmet need for family planning in Benin. Global Public Health.

[bib47] International Institute for Population Sciences (IIPS), ICF (2017). National family health survey (NFHS-4), 2015-16: India.

[bib48] International Institute for Population Sciences (IIPS) and Macro International (2007).

[bib49] Islam M.K., Haque M.R., Hossain M.B. (2016). Regional variations in child marriage in Bangladesh. Journal of Biosocial Science.

[bib50] Jacob S. (2015). Towards a comparative subnational perspective on India. Study of Indian Politics.

[bib51] Jha J., Minni P., Priya S.T., Chatterjee D. (2016). Reducing child marriage in India: A model to scale up results.

[bib52] Kalamar A.M., Lee-Rife S., Hindin M.J. (2016). Interventions to prevent child marriage among young people in low- and middle-income countries: A systematic review of the published and gray literature. Journal of Adolescent Health.

[bib53] Kaur R., Palriwala R. (2018). Marrying in south Asia: Shifting concepts, changing practices in a globalising world.

[bib54] Lapinski M.K., Rimal R.N. (2005). An explication of social norms. Communication Theory.

[bib55] Lee-Rife S., Malhotra A., Warner A., Glinski A.M. (2012). What works to prevent child marriage: A review of the evidence. Studies in Family Planning.

[bib56] LeSage J.P., Pace R.K. (2009). Introduction to spatial econometrics.

[bib57] LeSage J.P., Pace R.K., Fischer M.M., Nijkamp P. (2014). Interpreting spatial econometric models. Handbook of regional science.

[bib58] Liang M., Simelane S., Fortuny Fillo G. (2019). The state of adolescent sexual and reproductive health. Journal of Adolescent Health.

[bib59] Lo Forte C., Plesons M., Branson M., Chandra-Mouli V. (2019). What can the global movement to end child marriage learn from the implementation of other multi-sectoral initiatives?. BMJ Global Health.

[bib7] Mackie G., Moneti F., Shakya H., Denny E. (2015). What are Social Norms? How are they Measured?.

[bib60] Maiga A., Hounton S., Amouzou A. (2015). Trends and patterns of modern contraceptive use and relationships with high-risk births and child mortality in Burkina Faso. Global Health Action.

[bib61] Malhotra A., Warner A., McGonagle A., Lee-Rife S. (2011). Solutions to end child marriage: What the evidence shows.

[bib10] McDougal L., Jackson E.C., McClendon K.A., Belayneh Y., Sinha A., Raj A. (2018). Beyond the statistic: exploring the process of early marriage decision-making using qualitative findings from Ethiopia and India. BMC women's health.

[bib62] McPherson M., Smith-Lovin L., Cook J.M. (2001). Birds of a feather: Homophily in social networks. Annual Review of Sociology.

[bib63] Mehra D., Sarkar A., Sreenath P., Behera J., Mehra S. (2018). Effectiveness of a community based intervention to delay early marriage, early pregnancy and improve school retention among adolescents in India. BMC Public Health.

[bib64] Mennis J. (2006). Mapping the results of geographically weighted regression. The Cartographic Journal.

[bib65] Ministry of Women and Child Development, Government of India (2010). Approval of Rajiv Gandhi Scheme for empowerment of adolescent girls (RGSEAG) - SABLA.

[bib66] ML Infomap (2011). Subdistrict map of India : Digital map of subdistrict boundaries of India 2011.

[bib67] Nguyen P.H., Frongillo E.A., Kim S.S. (2019). Information diffusion and social norms are associated with infant and young child feeding practices in Bangladesh. Journal of Nutrition.

[bib68] Population Division of UN Department of Economic and Social Affairs (UNDESA) (2017). 2017 revision of world population prospects.

[bib69] Prakash R., Beattie T.S., Javalkar P. (2019). The Samata intervention to increase secondary school completion and reduce child marriage among adolescent girls: Results from a cluster-randomised control trial in India. Journal of Global Health.

[bib3] Raj A. (2010). When the mother is a child: the impact of child marriage on the health and human rights of girls. Arch Dis Child.

[bib70] Reno W. (2013). The problem of extraterritorial legality. Interview Research in Political Science.

[bib3a] Raj A., Saggurti N., Lawrence D., Balaiah D., Silverman J.G. (2010). Association between adolescent marriage and marital violence among young adult women in India. Int JGynaecol Obstet.

[bib9] Raj A., Ghule M., Nair S., Saggurti N., Balaiah D., Silverman J.G. (2015). Age at menarche, education, and child marriage among young wives in rural Maharashtra, India. Int JGynaecol Obstet.

[bib12] Raj A., Salazar M., Jackson E.C. (2019). Students and brides: a qualitative analysis of the relationship between girls' education and early marriage in Ethiopia and India. BMC Public Health.

[bib71] Roest J. (2016). Child marriage and early child-bearing in India: Risk factors and policy implications.

[bib5] Shakya H.B., Christakis N.A., Fowler J.H. (2014). Association Between Social Network Communities and Health Behavior: An Observational Sociocentric Network Study of Latrine Ownership in Rural India. Am J Public Health.

[bib6] Shakya H.B., Christakis N.A., Fowler J.H. (2015). An exploratory comparison of name generator content: data from rural India. Working Paper.

[bib11] Shakya H.B., Mackie G., Nkwi P., Cislaghi B. (2018). Social Norms Sustaining Child Marriage in Cameroon: A Qualitative Report In.

[bib14] Shakya H.B., Weeks J.R., Christakis N.A. (2019). Do village-level normative and network factors help explain spatial variability in adolescent childbearing in rural Honduras?. SSM Popul Health.

[bib15] Shakya H.B., Weeks J., Challa S. (2020). Spatial analysis of individual- and village-level sociodemographic characteristics associated with age at marriage among married adolescents in rural Niger. BMC Public Health.

[bib72] Sankaran M. (2005). Micro credit in India: An overview. World Review of Entrepreneurship Management and Sustainable Development.

[bib73] Santhya K.G., Ram U., Acharya R., Jejeebhoy S.J., Ram F., Singh A. (2010). Associations between early marriage and young women's marital and reproductive health outcomes: Evidence from India. International Perspectives on Sexual and Reproductive Health.

[bib74] Sedlander E., Rimal R.N. (2019). Beyond individual-level theorizing in social norms research: How collective norms and media access affect adolescents' use of contraception. Journal of Adolescent Health.

[bib75] Shiferaw S., Abdullah M., Mekonnen Y. (2015). Trends in contraceptive use and distribution of births with demographic risk factors in Ethiopia: A sub-national analysis. Global Health Action.

[bib76] Shulman H.C., Levine T.R. (2012). Exploring social norms as a group-level phenomenon: Do political participation norms exist and influence political participation on college campuses?. Journal of Communications.

[bib77] Srinivasan P., Khan N., Verma R., Guisti D., Theis J., Chakraborty S. (2015). District-level study on child marriage in India: What do we know about the prevalence, trends and patterns?.

[bib78] Srinivasulu K. (2002). Caste, class and social articulation in Andhra Pradesh: Mapping differential regional trajectories.

[bib79] Starmann E., Heise L., Kyegombe N. (2018). Examining diffusion to understand the how of SASA!, a violence against women and HIV prevention intervention in Uganda. BMC Public Health.

[bib80] Su X. (2018). Fragmented sovereignty and the geopolitics of illicit drugs in northern Burma. Political Geography.

[bib81] Svanemyr J., Amin A., Robles O.J., Greene M.E. (2015). Creating an enabling environment for adolescent sexual and reproductive health: A framework and promising approaches. Journal of Adolescent Health.

[bib8] Svanemyr J., Chandra-Mouli V., Raj A., Travers E., Sundaram L. (2015). Research priorities on ending child marriage and supporting married girls. Reprod Health.

[bib82] Swain R.B., Wallentin F.Y. (2009). Does microfinance empower women? Evidence from self-help groups in India. International Review of Applied Economics.

[bib83] Times of India (2017). In Hadauti, brides are available for Rs 50,000.

[bib84] UNFPA, UNICEF (2018). 2017 annual report - accelerating and amplifying change: UNFPA-UNICEF global programme to accelerate action to end child marriage.

[bib85] UNFPA, UNICEF (2018). 2017 annual report country profiles.

[bib86] UNICEF Data and Analytics Section (2018). Progress for every child in the SDG era: Are we on track to achieve the SDGs for children?.

[bib87] UNICEF-UNFPA (May 2019). UNFPA-UNICEF global programme to accelerate action to end child marriage: Joint evaluation report.

[bib88] United Nations (2020). Policy brief: The impact of COVID-19 on children. 15 April 2020.

[bib89] Verma R., Sinha T., Khanna T. (2013). Asia child marriage initiative: Summary of research findings in Bangladesh, India and Nepal.

[bib90] Vindhya U., Dev V.S. (2011). Survivors of sex trafficking in Andhra Pradesh: Evidence and testimony. Indian Journal of Gender Studies.

[bib91] Wakefield M.A., Loken B., Hornik R.C. (2010). Use of mass media campaigns to change health behaviour. Lancet.

[bib92] Weeks J.R., Goodchild M.F., Janelle D.G. (2004). The role of spatial analysis in demographic research. Spatially integrated social science: Examples in best practice.

[bib93] Weeks J.R., FM H., JR P., SA M. (2015). Demography is an inherently spatial science. Recapturing space: New middle-range theory in spatial demography.

[bib94] Weeks J.R., Getis A., Hill A.G., Agyei-Mensah S., Rain D. (2010). Neighborhoods and fertility in Accra, Ghana: An AMOEBA-based approach. Annals of the Association of American Geographers.

