# Supplementary material for: Mapping the patchwork: Exploring the subnational heterogeneity of child marriage in India
Source: SSM Popul Health. 2020 Nov 16;12:100688. doi: 10.1016/j.ssmph.2020.100688 (PMC7726340; doi:10.1016/j.ssmph.2020.100688)
Supplement: Multimedia component 1 [file mmc1.docx]

**Appendix**

| **Sociodemographics** | |
| --- | --- |
| Rural residence | |
| A. | B. |
| 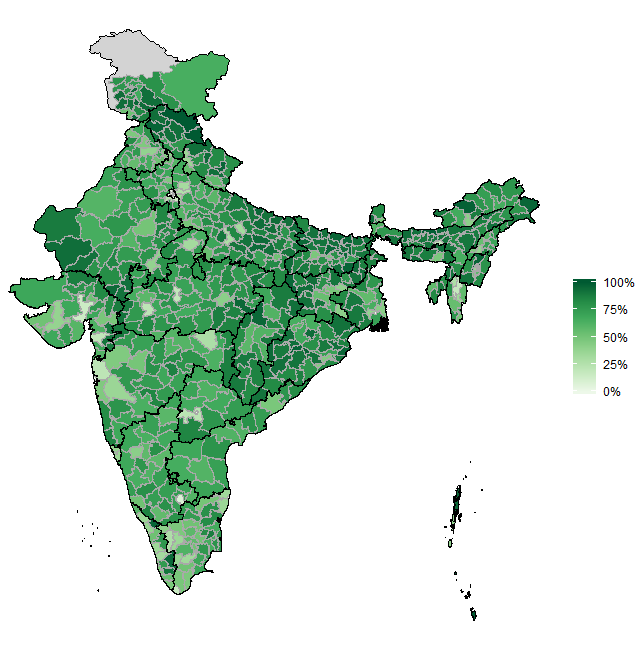 | 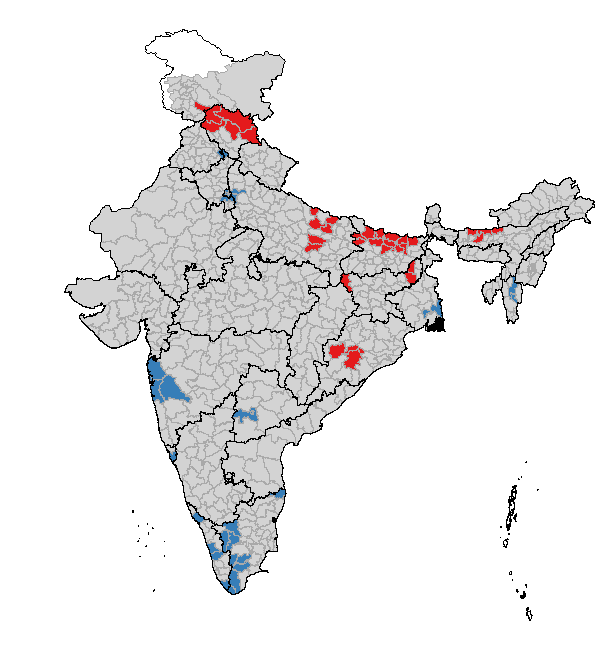 |
| SC/ST or OBC | |
| A.  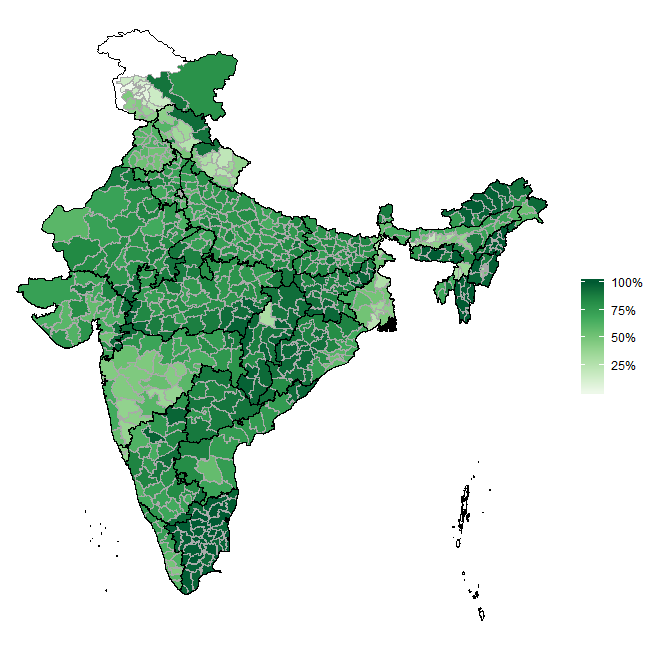 | B.  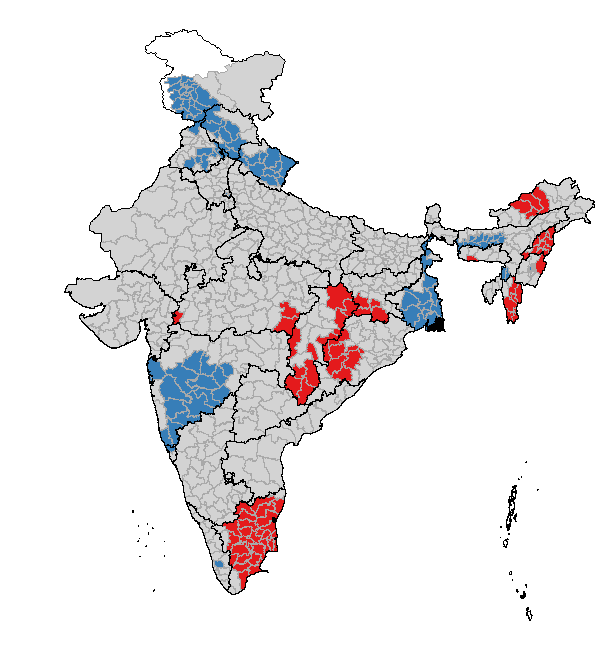 |
| Muslim | |
| A.  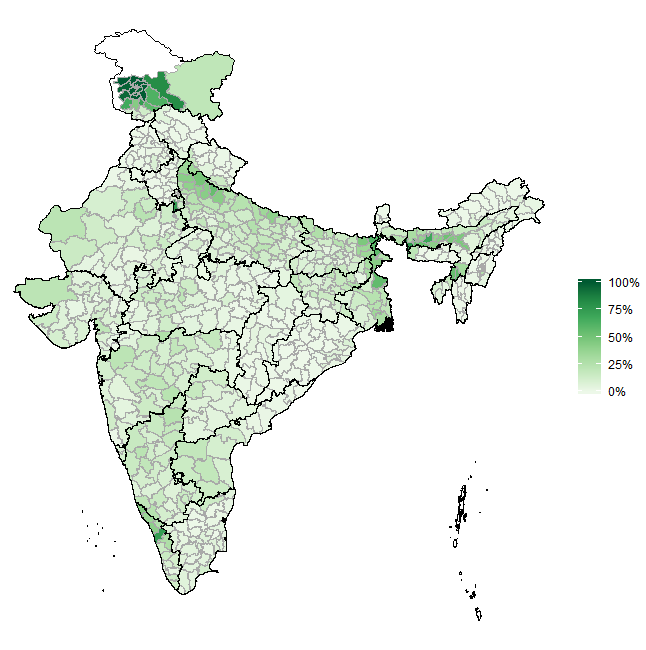 | B.  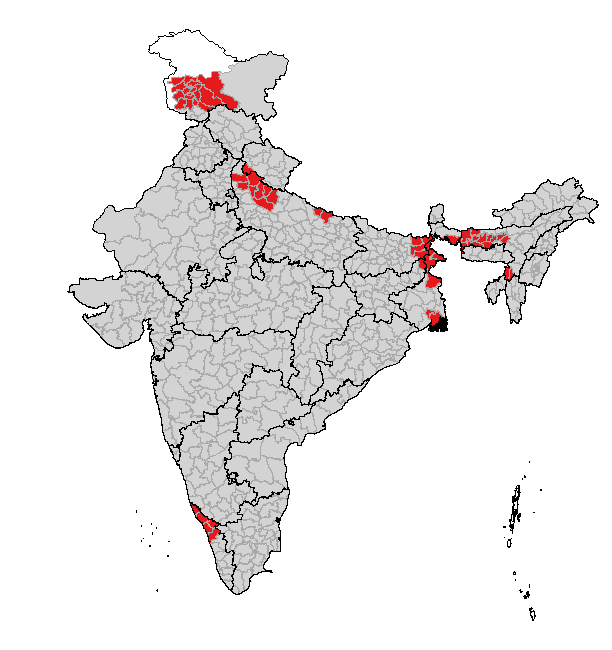 |
| Female education | |
| A. | B. |
| 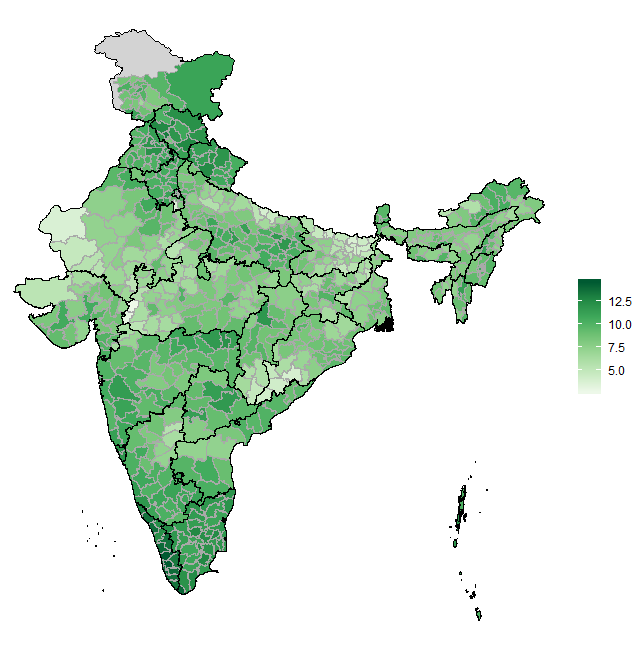 | 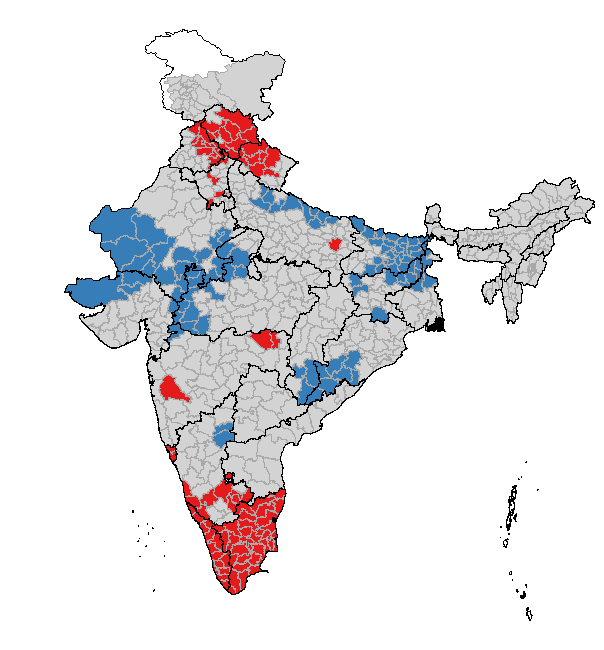 |
| Female:male sex ratio at birth | |
| A. | B. |
| 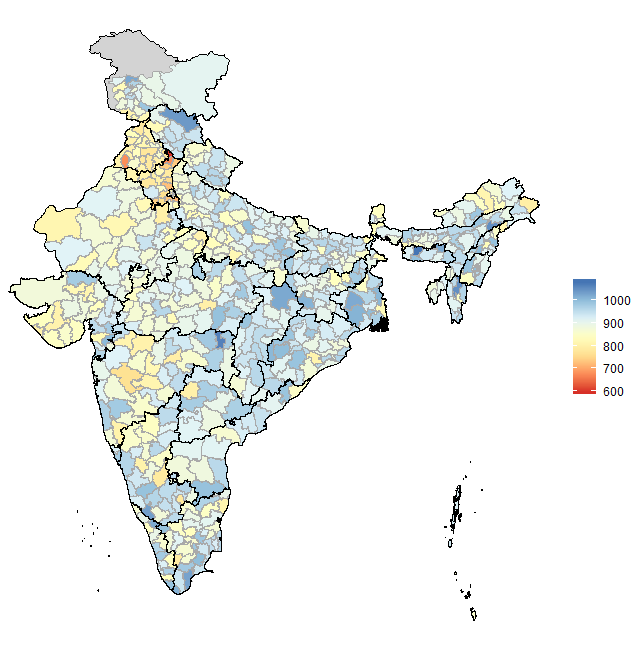 | 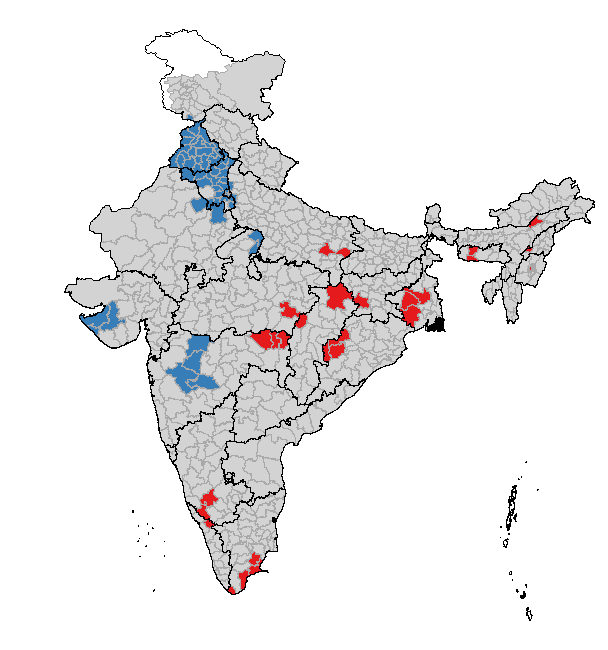 |
| District-state differences in prevalence of child marriage | |
| A. | B. |
| 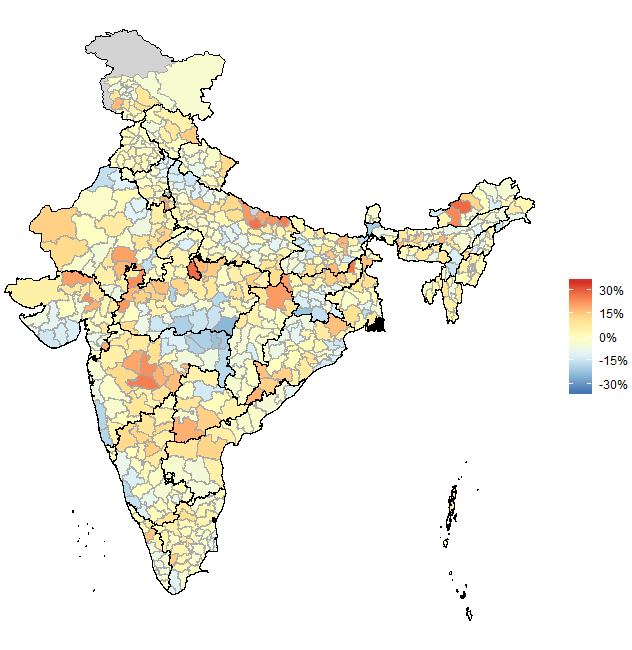 | 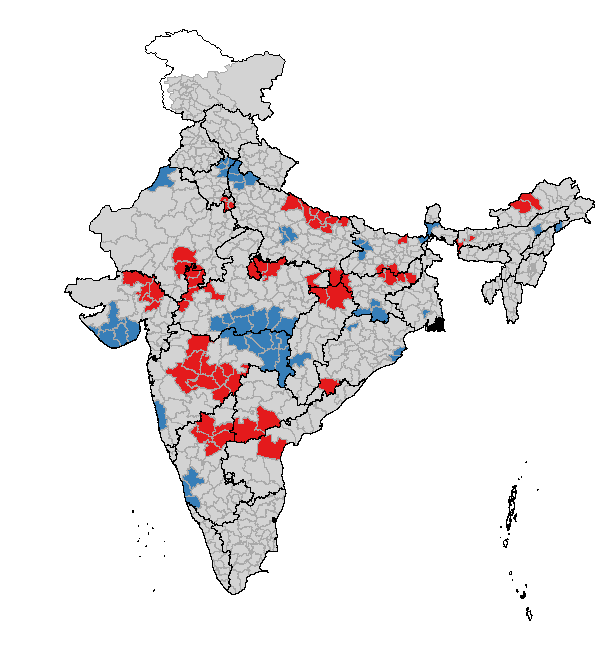 |
| **Media connectivity** | |
| Female television use | |
| A. | B. |
| 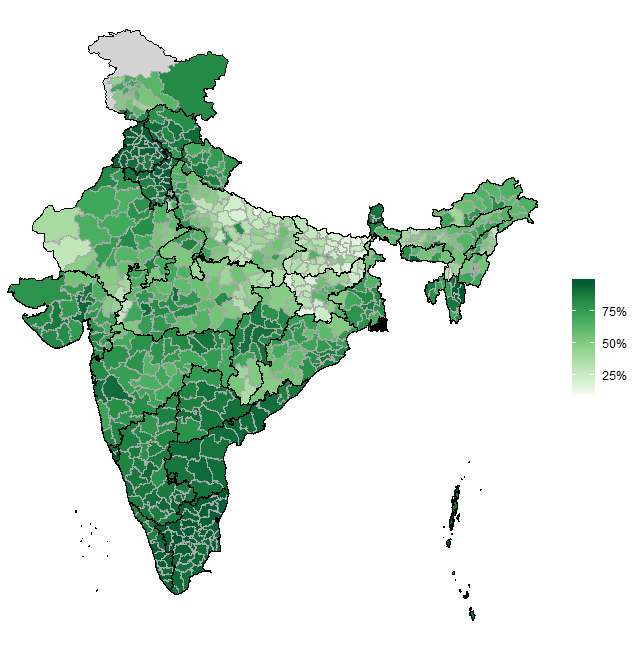 | 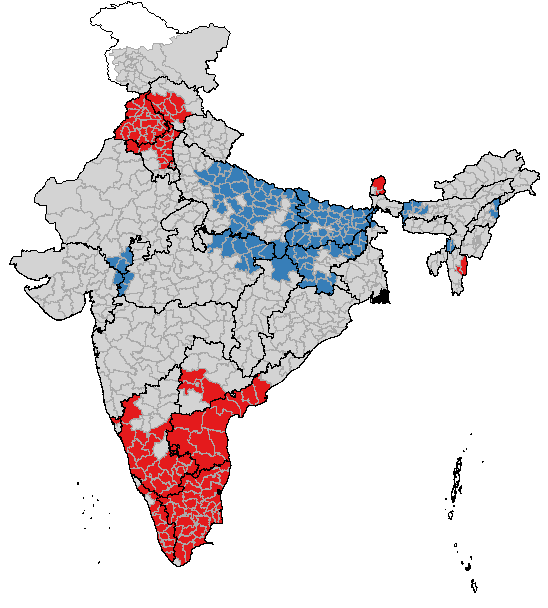 |
| Female radio use | |
| A. | B. |
| 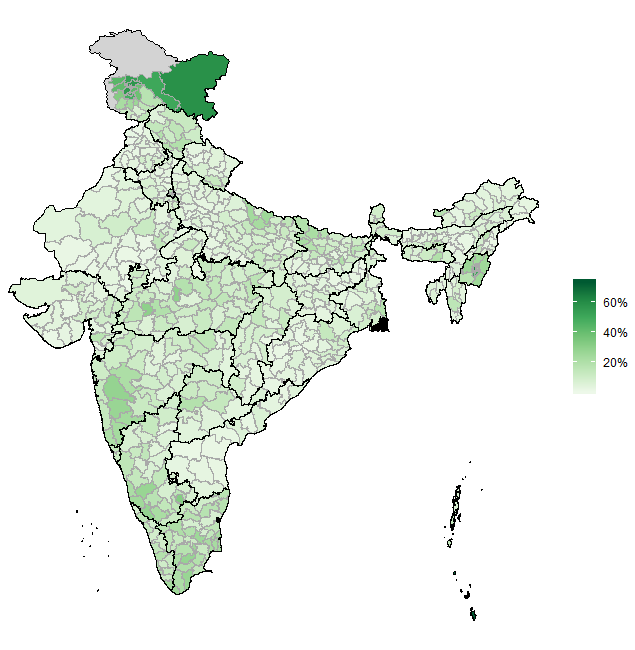 | 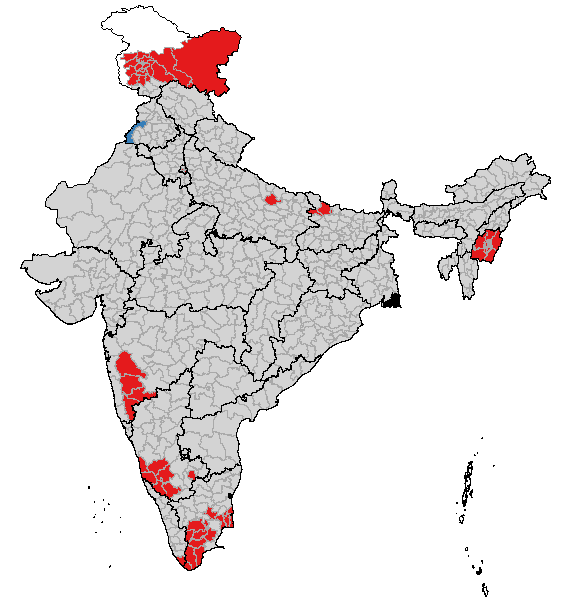 |
| Female newspaper use | |
| A. | B. |
| 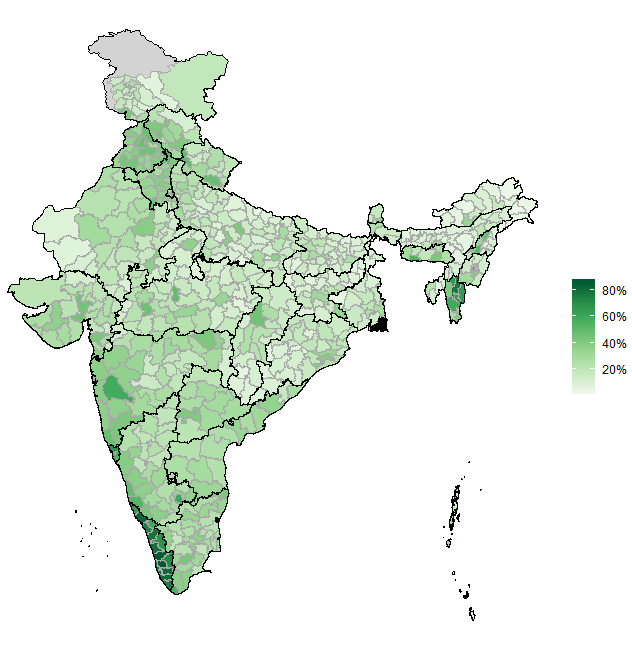 | 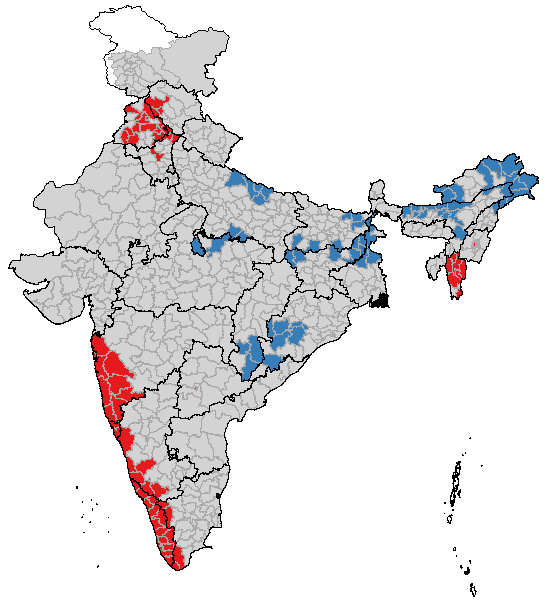 |
| Female mobile phone access | |
| A. | B. |
| 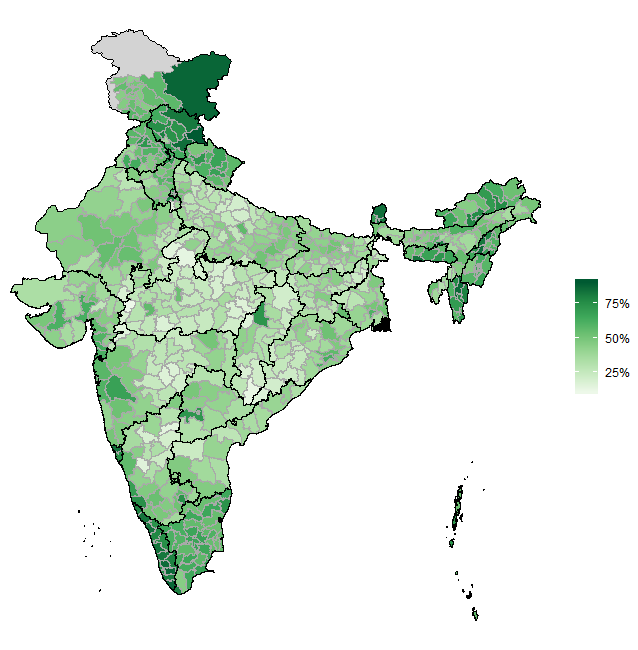 | 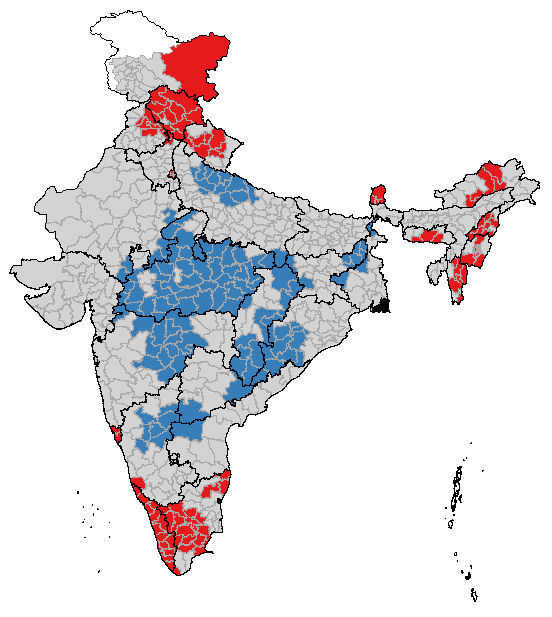 |
| Households with internet access | |
| A. | B. |
| 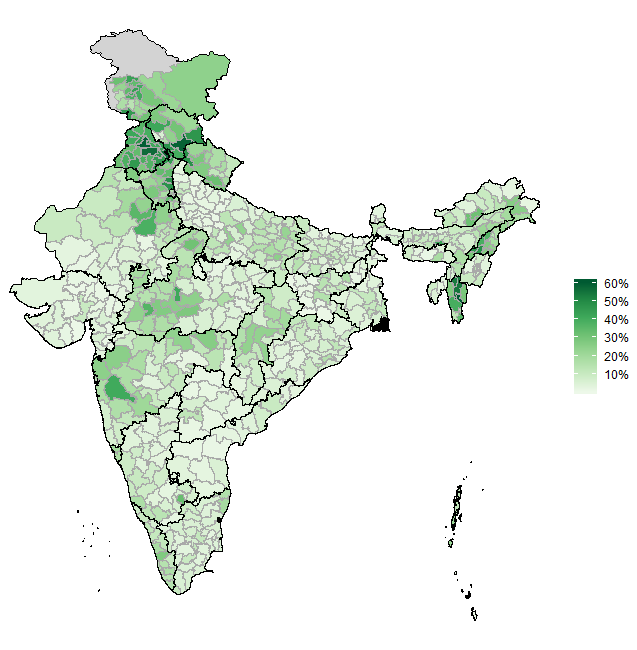 | 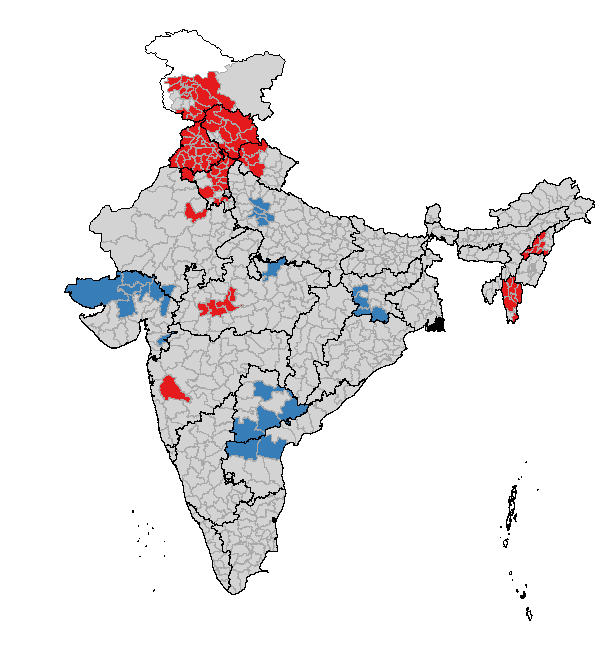 |
| **Community connectivity** | |
| Female microcredit program awareness (%) | |
| A. | B. |
| 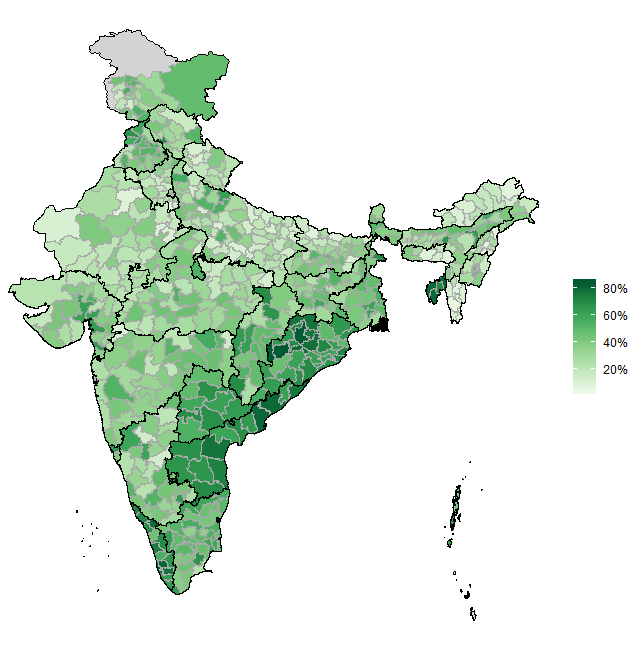 | 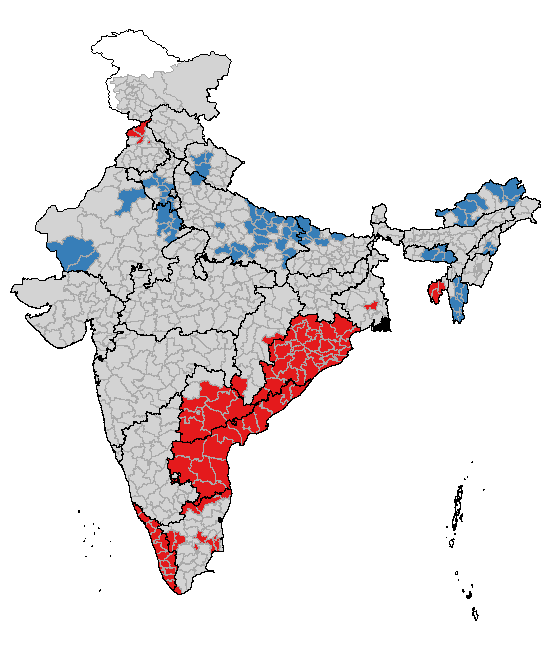 |
| Female microcredit program utilization (%) | |
| A.  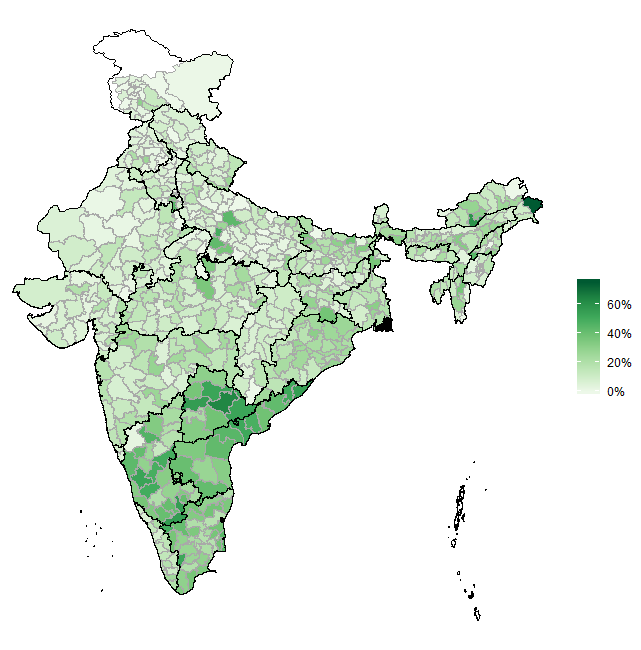 | B.  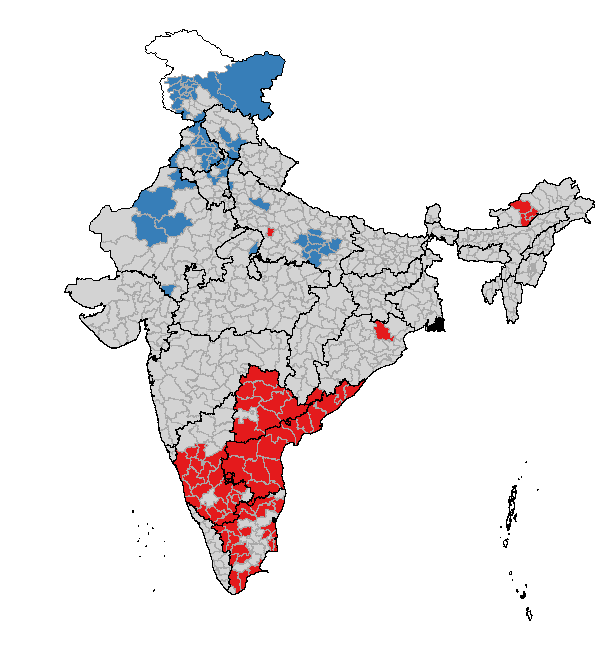 |

Appendix Figure 1. Prevalence (A) and local measures of spatial autocorrelation (B) of assessed sociodemographic and media connectivity variables in 2015-16.

Note: In prevalence maps (A), white represents data not available. In LISA maps (B), red indicates hot spots, and blue indicates cold spots (clusters of districts with high and low [respectively] prevalences that are statistically similar to their neighbors at p<0.05). Grey indicates no significance.
